# Advertising Unofficial Preparations

**Published:** 1908-04

**Authors:** 


### Abstracts and Selections.

### Advertising Unofficial Preparations.

The American Medical Association—or rather the bureau which *controls* the Association, for it can not be supposed that the members at large had any concern in the matter beyond the perfunctory passage of any resolutions presented to them from the council chamber—has recently communicated to the medical press of the country a resolution adopted at its last annual convention, requesting them to refuse advertisements of all unofficial remedies which have not yet been passed upon by the Council on Chemistry and Pharmacy and assigned a place in its blue book.

It is unnecessary to reopen at this time the whole subject of proprietary remedies and the jurisdiction of the Council on Chemistry and Pharmacy. Undoubtedly the communication referred to will carry to the society organs in the various States the expression of a more of less peremptory command, which we, nevertheless, venture to predict will not be any too broadly observed. For the rest, it is enough to point out that there are many and excellent reasons why the independent medical press can not and should not comply with a request of this gratuitous and unreasonable character.

It might, indeed, occur to any intelligent person, as a foregone and axiomatic reply to such a propaganda, that the independent medical press, in virtue of the essential nature and quality of its function, is estopped from complying with the request. It is like asking an employer who is, as a matter of principle, contending for the open shop, to treat with the union. The independent medical press has obligations which it is bound to fulfill, no less than the society organ—obligations more sacred and binding because more fundamental. It stands distinctively for the independent practitioner, *whether inside or outside of the union,* who, while he approves of organization for purposes of scientific and economic progress, *denies both the right and the expediency of any organized paternalism, and resents any attempted curtailment of his individual judgment.*

But supposing that we waive the question of unionism, and discuss the matter sheerly upon its merits; the result will not be widely different. Granting, for the nonce, the value and validity of the Council's function, is it wise at this time to engage in a concerted boycott of all those pharmacal preparations which thus far failed to undergo its investigation and to receive its approval?

In the first place, the number of preparations already passed upon by the Council is exceedingly small. It can not for a moment be assumed that all the rest are unworthy. Does the Association really and seriously suggest that all preparations—many of which are in every-day clinical use and favor by thousands of practicing physicians throughout the country—shall be arbitrarily kept in the antechamber cooling their heels and awaiting the the convenience of the self-constituted grand jury? And if so, *cui bono?* It does not even appear that the Council is provided with, or is employing, any clinical methods of trying out the preparations under investigation. The fact is—and this affords a further powerful argument against acceding to the Association's ill-advised request—it has already examined and rejected more than one unofficial remedy of whose clinical worth, despite the Council's erudite criticism, there is no doubt in the minds of thousands of able and honest practitioners of medicine. Does the Association really and seriously expect that the verdict of the Council upon these preparations will finally dispose of them in the face of their almost universal clinical endorsement?

But far more fundamental than any of the considerations thus far mentioned is the baneful and mischievous effect which such a concerted action on the part of the medical press of the country would have upon pharmacal and therapeutic progress. Scarcely any proposition could be formulated which would more powerfully demonstrate the pernicious tendencies of an over-organized medical press, and the imperative necessity of the independent journal. Specialized investigation is a very excellent thing in its proper province and at its proper valuation. But there never has been an instance of its playing any effective part in permanent evolution or progress, and in this respect it can never supplant, nor will it ever again replace, the more trustworthy process of natural selection. The adoption of such a plan as that suggested by the Association would set the matter of pharmaceutic commodities back where pharmaceutic knowledge was in the Middle Ages. It would establish an index purgatorious of pharmaceutical preparations subject to the papal autocracy of the Council on Pharmacy. Imagine a condition of similar restriction upon the dissemination of scientific knowledge, and figure how much progress science would be likely to make under such a prohibition. Already the free discussion of proprietary preparations in the editorial pages of medical journals is tabooed as an undesirable proceeding; only the very bravest of the independent journals dare to engage in it. Now it is proposed to cut off the economic channels by which the products of pharmaceutic skill may freely reach the medical man for his own unhampered trial and judgment. *Can anyone seriously believe that such a course is for the best interests of medical science or anyone connected with it?*

It must not be supposed, because this is our attitude, that we, therefore, advocate the throwing open of the independent advertising columns to any and every preparation that is offered, provided only that it pays the price of the space. We anticipate just such an alleged alternative in the thoughts, if not in the words of our extremely ethical (?) friends; and, indeed, it has already more than once found expression in their criticisms. But is not well chosen, and displays a lack of discrimination. On the contrary, we regard ourselves, in our journalistic capacity, as a responsible factor in that true evolution of medical science and medical technique which we have characterized as natural selection. But we do not regard ourselves as the omnipotent arbiter of such evolution. We believe it to be the function of a medical journal to provide in its editorial and its advertising "things honest in the sight of all men," and to use its reasonable care and judgment in so doing.

If we offered, for our editorial pages, an article or a report, emanating from a scientific man, which bears upon its face the ordinary evidence of good faith and honesty, and which, if true, is of value to the practitioner we publish it. To refuse to give it place until its absolute value had been permanently established would put a stop to all scientific progress, so far as our efforts were concerned. It is for the medical public, to whom it is offered, to "try the spirits." In the matter of pharmaceutical armamentaria, made public through our advertising pages, the same principle applies. The exercise of reasonable judgment in the discrimination of ordinary good faith and honesty and of probable value to the profession is, in our judgment, the only legitimate part for the medical journal to play in the process of natural selection by which the good is garnered and the useless rejected. The ultimate determination of permanent values is not the function of any journal, nor the Council on Chemistry and Pharmacy, nor any other institution, but of the profession at large.—*Medical Brief.*

